# An open-source nanopore-only sequencing workflow for analysis of clonal outbreaks delivers short-read level accuracy

**DOI:** 10.1128/jcm.00664-25

**Published:** 2025-07-18

**Authors:** Nick Vereecke, Thomas B. Yoon, Ting L. Luo, Brendan W. Corey, Francois Lebreton, Patrick T. Mc Gann, John P. Dekker

**Affiliations:** 1 Bacterial Pathogenesis and Antimicrobial Resistance Section (BPARS), Laboratory of Clinical Immunology & Microbiology (LCIM), National Institute for Allergy and Infectious Disease (NIAID), National Institutes of Health (NIH) 35037 , Bethesda, Maryland, USA; 2 Multidrug-Resistant Organism Repository and Surveillance Network (MRSN), Diagnostics and Countermeasures Branch, Center for Infectious Disease Research (CIDR), Walter Reed Army Institute of Research (WRAIR) 8394, Silver Spring, Maryland, USA; Maine Medical Center Department of Medicine, Portland, Maine, USA

**Keywords:** long-read nanopore sequencing, epidemiology, bioinformatics, error correction, genome sequencing, open-source, bacteriology, clinical microbiology, public health microbiology, outbreak detection, cgMLST, minimum spanning tree

## Abstract

In this work, we present an optimized nanopore long-read only sequencing workflow for epidemiologic analysis of clonal outbreaks built with open-source tools. A set of unrelated clinical *Pseudomonas aeruginosa* isolates (*n* = 10) was chosen for workflow optimization, and sequencing libraries were prepared using a modified rapid barcoding strategy that incorporates temperature ramps to improve performance for high-GC content genomes. Sequencing data were used to benchmark the performance of the dorado suite (v0.9.1), including its basecaller, pre-assembly read error correction, and post-assembly polishing algorithms. All long-read assemblies and core genome multilocus sequence typing (cgMLST) were performed with Flye and pyMLST, respectively. Results were compared with a standard reference Illumina short-read approach, and discordant positions were determined at the core and whole-genome levels. Optimal performance was found with dorado sup@v5.0.0 basecalling with the inclusion of dorado error correction and dorado polish with its bacterial model. This workflow was then validated with four retrospective hospital outbreak isolate sets, including *Klebsiella pneumoniae* (*n* = 12), *P. aeruginosa* (*n* = 11), *Enterococcus faecium* (*n* = 10), and *Staphylococcus aureus* (*n* = 10). The nanopore-only assemblies obtained from the optimized pipeline demonstrated fully concordant cgMLST-based minimum spanning trees compared to the Illumina short-read reference. At the whole-genome level, high concordance was also observed, with as few as two discordant positions per genome compared to short-read assemblies. This optimized library preparation and open-source computational workflow enables nanopore-only clonality and outbreak analysis with performance comparable to that of Illumina short-read sequencing and will contribute critically to hospital infection control.

**IMPORTANCE:**

For the past decade, bacterial whole-genome sequencing has been performed using high-accuracy short-read sequencing. More recently, long-read sequencing with Oxford Nanopore Technologies (ONT) instruments has emerged as a potential alternative based on multiple advantages, including lower costs, portability, and speed. However, this platform has suffered from basecall error rates that were too high for many applications in clinical microbiology, including outbreak tracing. With the release of new flow cell chemistries and basecall algorithms, the accuracy has improved dramatically, making this approach feasible for outbreak investigations. In this work, we optimize a streamlined nanopore-only workflow for epidemiologic analysis of bacterial pathogens. The workflow was validated with isolates from four previously identified clinical outbreaks with varying GC content and demonstrated fully concordant cgMLST clustering as compared to short-read references. This workflow will facilitate the broader implementation of ONT-only genomes and cgMLST analysis to assist in hospital outbreaks worldwide.

## INTRODUCTION

Genomic sequencing has become a standard component of epidemiologic investigations, where it plays an indispensable role in establishing clonality and relatedness of bacterial isolates involved in a transmission cluster or outbreak (1, 2). Commonly used genomic approaches in such investigations include core genome multilocus sequence typing (cgMLST), core genome single-nucleotide polymorphism (cgSNP), and whole-genome single-nucleotide polymorphism (wgSNP) analysis. The cgMLST approaches are similar to the original MLST methods, in which allelic differences are used to infer genetic distances, though cgMLST methods are applied across hundreds to thousands of core genes, allowing finer resolution analysis of relatedness (3–10). Alternatively, cgSNP and wgSNP approaches compute distances based on counts of all variants, as opposed to allelic counts, and may have certain advantages in some contexts (11, 12). Implementation of these genomic approaches in hospital, clinical microbiology, and public health laboratories has been made possible by bioinformatic tools that are available under license agreement or as open-source (13, 14).

For the past decade, cgMLST, cgSNP, and wgSNP analyses have been largely performed using high-accuracy short-read sequencing (SRS) approaches. Long-read sequencing (LRS) with Oxford Nanopore Technologies (ONT) instruments has emerged as a potential alternative based on multiple advantages, including lower instrument costs, portability, and speed (15). However, ONT nanopore sequencing has historically suffered from basecall error rates that have been too high for many applications in clinical microbiology and epidemiology, including determination of clonality for outbreak analysis. Various long-read cgMLST workflows have been proposed in recent years, including the use of hybrid sequencing that combines SRS and LRS (15), taxon-specific approaches (16–18), PCR-based library preparations to remove errors associated with bacterial methylation events (19), and proprietary polishing tools (20). Despite these advances, the error rates have remained above acceptable levels, resulting in the lack of broader implementation of ONT-only workflows. This situation has changed considerably with the release of the R10.4.1 flow-cell chemistry and improved basecalling models, markedly increasing accuracy and making nanopore-only epidemiologic applications conceivable (1, 15, 16, 20–25).

A variety of approaches have been used to improve the accuracy of long-read consensus genome assemblies, including pre-assembly raw read error correction (26), taxon-specific basecall models (23, 25), combinations of assembly algorithms (24, 27), and post-assembly genome polishing (e.g*.,* pilon [28], racon [29], and medaka (ONT)). Raw read error correction has gained new attention with the recent introduction of the novel HERRO deep learning algorithm (30). This approach is available in the latest release of the dorado suite (dorado correct; v0.9.1; ONT), along with the new dorado polish for post-assembly polishing, which is suggested to replace ONT’s medaka polisher (31, 32).

In this work, we optimize an open-source ONT-only workflow for epidemiologic analysis of clonal outbreaks and transmission of bacterial pathogens with a focus on accessibility and performance. We additionally test an approach for library preparation that incorporates a temperature ramp to improve performance on high-GC content genomes, using *P. aeruginosa* as a model test organism. We demonstrate the concordance between an Illumina reference method and this ONT-only approach for generating cgMLST-based minimum spanning trees. This work demonstrates that nanopore-only clonality and outbreak analysis can be performed, with performance comparable to that of Illumina short-read sequencing and will contribute critically to hospital infection control.

## RESULTS

### Improved sequencing throughput for high GC *P. aeruginosa* DNA using tagmentation with temperature ramps

**Fig 1:**
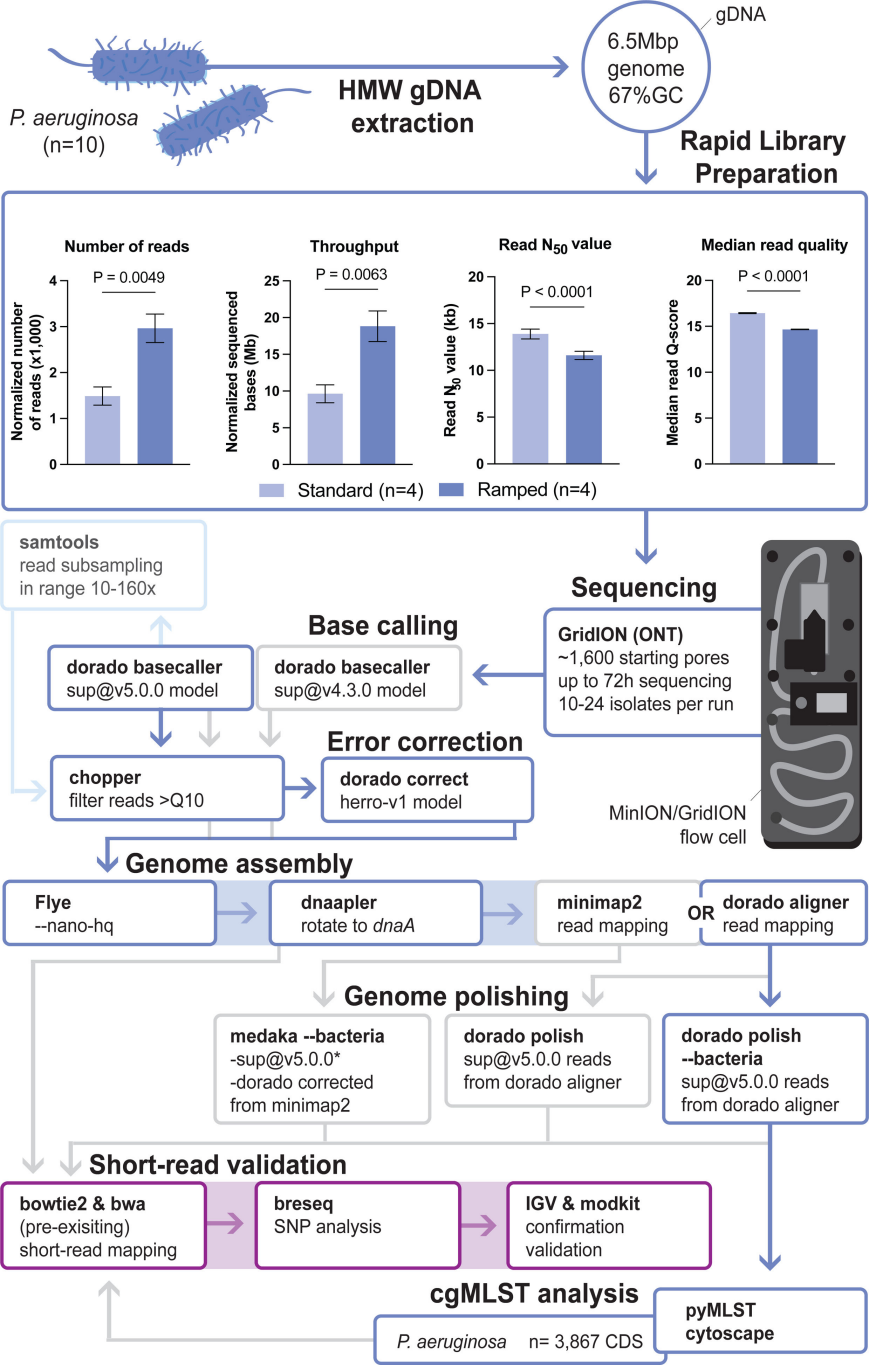
Schematic overview of benchmarking approach, highlighting the optimized pipeline. A diverse test set of *P. aeruginosa* (*n* = 10) isolates was used for HMW gDNA extraction, followed by SQK-RBK114.24 long-read sequencing library preparation. The rapid library preparation included a comparison of the standard rapid barcoding protocol with a temperature-ramped tagmentation protocol for high-GC *P. aeruginosa* test samples (*n* = 4 in duplicate barcodes; *n* = 8), as sequenced on R10.4.1 Flongle flow cells and basecalled within MinKNOW (dorado with sup@4.3.0). A greater number of normalized reads and increased sequencing throughput were observed (see Methods for normalization), along with decreased N_50_ read length values and median quality scores. Mean ± s.e.m. are presented for each parameter, and the significance was determined using Student’s *t*-test and is shown on the plots with *P* values. The temperature ramp protocol was evaluated using Flongle flow cells. Sequencing of the *P. aeruginosa* data set was performed with an R10.4.1 GridION/MinION flow cell. Resulting sequencing data were subjected to various genome assembly pipelines, including (i) different dorado basecalling models; (ii) read filtering using chopper; (iii) read error correction (herro-v1 model); (iv) genome assembly using Flye; and finally genome polishing using either medaka or dorado polish with relevant models. The output of each ONT bioinformatics workflow was assessed through comparison with short-read data sets using breseq. Discordant positions were confirmed and validated in IGV (see Methods for classification of discordant positions). The final optimized pipeline indicated in dark blue was used for cgMLST analysis with open-source pyMLST and Cytoscape. *Legacy sup@v.4.3.0 reads were used to polish its respective genome assembly.

Reduced LRS rapid barcoding library efficiencies have been reported for high-GC content organisms, including *P. aeruginosa*, and thus we first aimed to increase the sequencing performance of four selected *P. aeruginosa* isolates from the test set (16, 21). Extracted high-molecular weight (HMW) DNA was subjected to duplicate rapid barcoding library preparations employing both a standard tagmentation approach and a protocol incorporating 0.1°C.s^−1^ temperature ramps (*n* = 8, methods). Inclusion of the temperature ramp resulted in a significantly greater number of normalized reads (1,487 ± 197 vs 2,966 ± 309; mean ± s.e.m), normalized sequencing throughput (9.6 ± 1.2 vs 18.8 ± 2.1 Mb), and a decrease in N_50_ read length values (13.9 ± 0.4 vs 11.6 ± 0.5 kb) (Fig. 1). Together, this suggests improved transposon cleavage using the ramped tagmentation protocol. A slight decrease in median raw read Q-scores (16.4 ± 0.1 to 14.7 ± 0.1 with dorado sup@4.3.0 basecalls within MinKNOW; ONT’s real-time basecalling and operating system) was also observed with ramped tagmentation and was not further investigated (Fig. 1). Given the overall improvement in the sequencing efficiency, a temperature-ramped rapid barcoding protocol was implemented for all *P. aeruginosa* test and validation isolates, while maintaining a standard non-ramped tagmentation for all other bacterial species in the validation data set.

### Dorado correct and polish result in highly accurate *P. aeruginosa* genomes

Next, we subjected the *P. aeruginosa* test data set to a benchmarking pipeline (Fig. 1 and Methods) to identify the LRS-only assemblies that were most concordant with the SRS reference. For this analysis, all identified discordant positions (i.e*.,* SNPs and indels) were manually evaluated using the exclusion criteria described in the Methods and summarized in Fig. S1. For nine of ten *P. aeruginosa* isolates, discordant analysis was performed using pre-existing SRS data that had been generated from prior DNA extractions (i.e*.,* not the same DNA used for LRS). For isolate PA288-2, SRS data were generated from the same HMW DNA used for LRS and used for discordant analysis.

**Fig 2:**
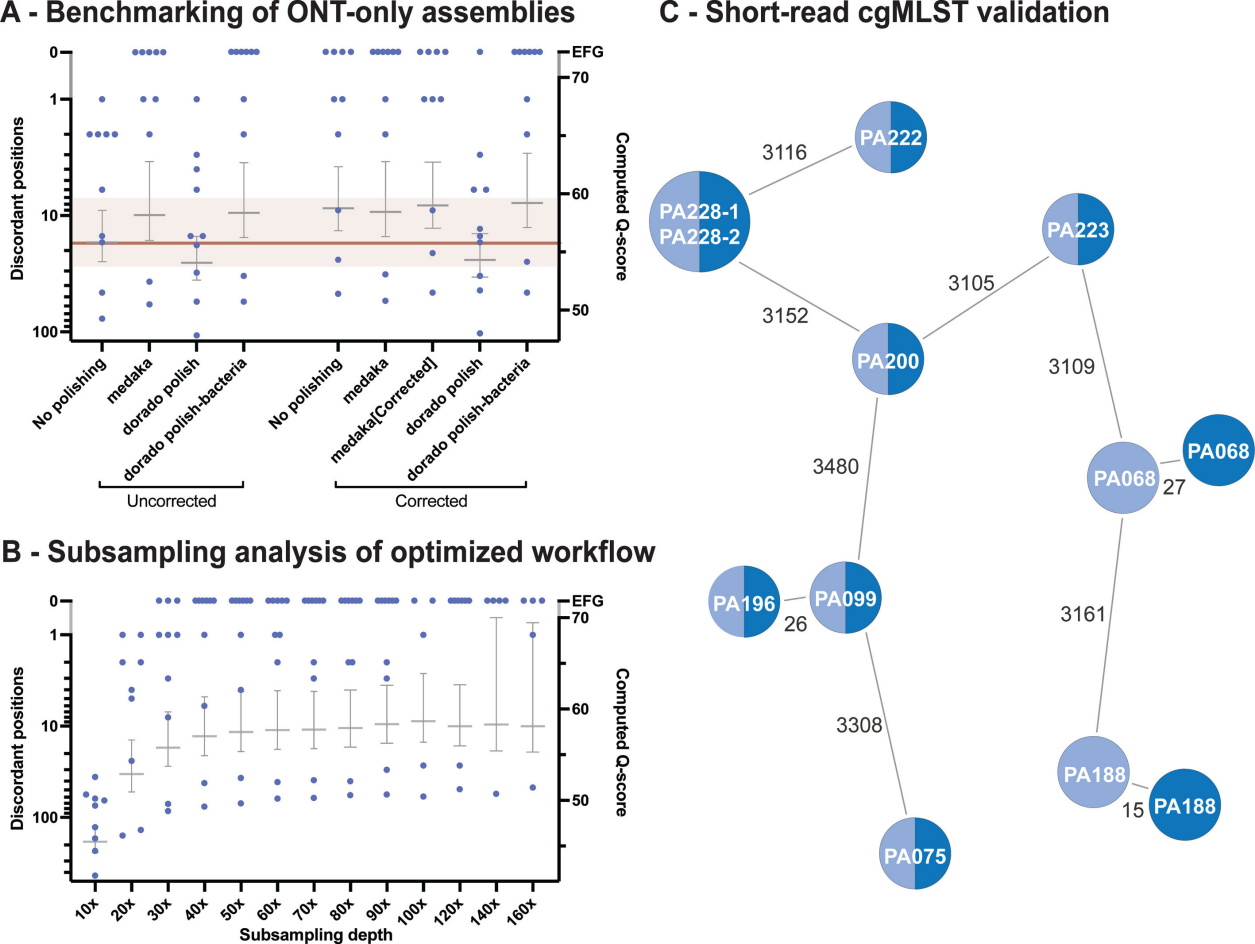
Benchmarking of the *P. aeruginosa* test data set, subsampling performance, and short-read cgMLST validation. (**A**) Chromosomal discordant positions (left y-axis) and computed Q-score (right y-axis) for different ONT-only *P. aeruginosa* assembly pipelines. The discordant positions were calculated based on respective Illumina short-read data sets. Red line and zone indicate mean ± s.e.m. for the legacy medaka-polished sup@v4.3.0 genomes. For each workflow, mean ± s.e.m. are presented with individual data points in blue. (**B**) Subsampling analysis of the test data set for the optimal workflow with subsampling at depths ranging from 10 up to 160 x. For each subsample, mean ± s.e.m. are presented with individual datapoints in blue. If a sample did not have sufficient coverage for a subsampling depth, it was excluded from the analysis. (**C**) Short-read cgMLST validation analysis to assess the impact of errors on cgMLST distances, as compared to Illumina short-read genome assemblies. Generation of minimum spanning trees was performed after short-read validation and classification of all discordant positions (Tables S3 and S5, and Methods), with branch lengths representing the number of allelic differences. Dark and light colors represent ONT and short-read data sets, respectively; EFG, error-free genomes based on Illumina references, corresponding to 0 discordant positions after applying exclusion criteria.

First, we assessed the performance of the latest sup@5.0.0 dorado basecalling model on the test set and compared its draft genomes with the legacy sup@4.3.0 genome assemblies, which were polished with medaka using the same sup@4.3.0 reads, as a baseline reference (red line in Fig. 2A and Methods). Unpolished Flye genome assemblies from sup@5.0.0 reads resulted in similar numbers of absolute discordant positions (and computed Q-scores) when compared to the medaka-polished sup@4.3.0 baseline (17 ± 8 vs. 17 ± 10 discordant positions; mean ± s.e.m; Fig. 2A; Table S3).

To assess the impact of dorado polishing on the improved sup@5.0.0 draft genomes, further genome polishing using either medaka or dorado polish with their respective bacterial models was compared. This resulted in a decrease in the number of discordant positions (10 ± 6 absolute discordant positions for both). Pre-assembly dorado correction of sup@5.0.0, followed by *de novo* Flye genome assembly, resulted in a comparable 9 ± 5 discordant positions without the need for additional polishing (Fig. 2A; Table S3). While polishing these drafts with medaka (using remapped corrected reads) or dorado polishing (with bacterial model using sup@5.0.0 reads) did further improve consensus genomes (8 ± 5 and 7 ± 5 discordant positions, respectively), medaka with remapped uncorrected reads (i.e*.,* sup@5.0.0 reads) retained a similar number of discordant positions (9 ± 6) (Fig. 2A; Tables S3 and S4). Use of the standard dorado polish model resulted in 26 ± 10 and 24 ± 10 discordant positions for Flye assemblies from uncorrected and corrected reads, respectively, and thus resulted in worse consensus genomes relative to the SRS reference, as compared to the baseline without dorado polish. On this basis, we chose the sup@v5.0.0 basecalled reads with dorado error correction and dorado polishing with its bacterial model to generate the most concordant bacterial genomes compared to the SRS data sets.

We also assessed the minimum required sequencing depth for the optimal workflow through a subsampling analysis on the same *P. aeruginosa* test data set. Subsampling at mean sequencing depths up to 160 x demonstrated that a 50 x mean depth was sufficient to create highly concordant genome assemblies (Fig. 2B; Table S3).

### Dorado correct and polish result in highly accurate cgMLST minimum spanning trees

**Fig 3:**
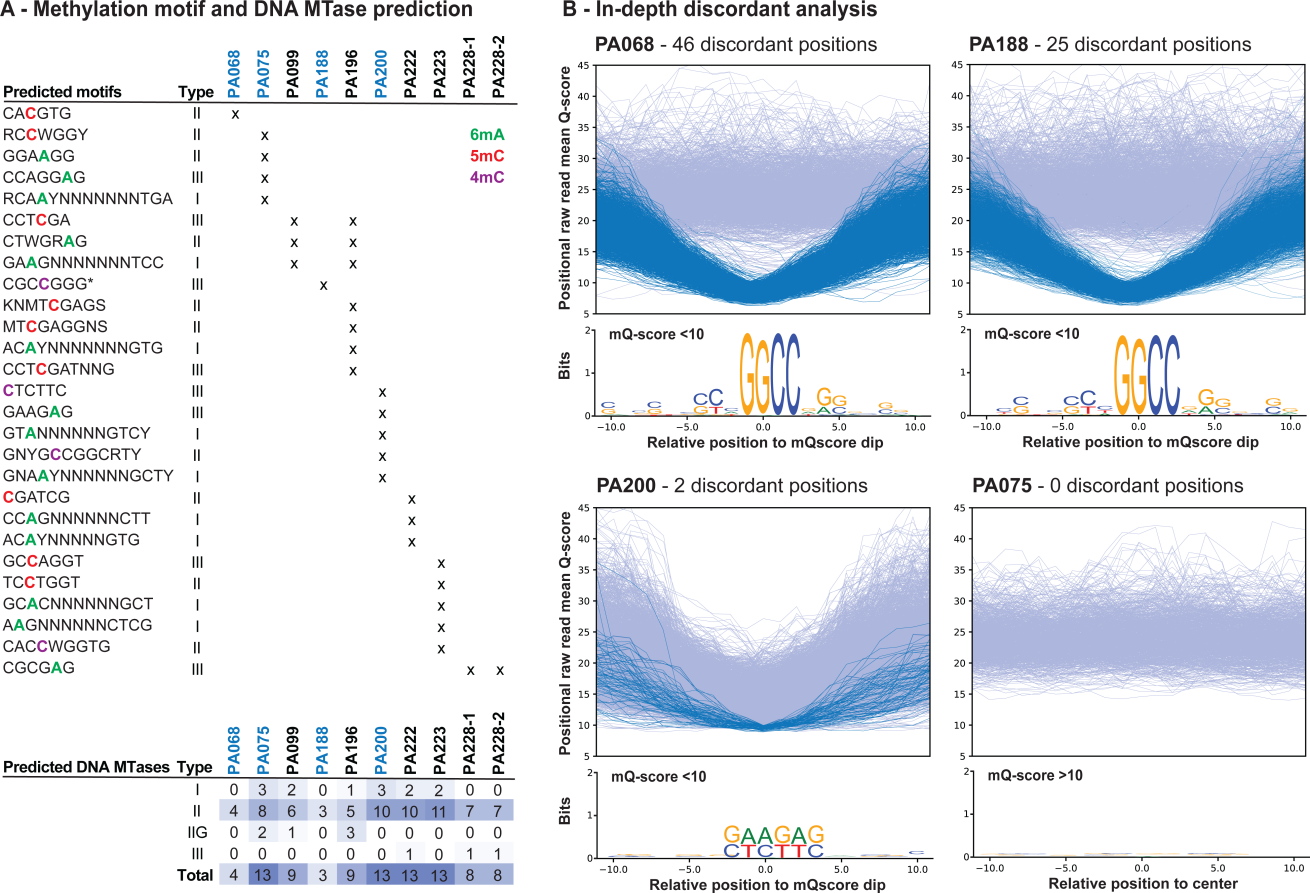
In-depth analysis of residual error profiles of *P. aeruginosa* test isolates. (**A**) For all *P. aeruginosa* test isolates (*n* = 10), methylation motifs were identified using the dorado basecaller methylation models (@v3 for 6mA and 4mC_5mC), followed by modkit analyses (Methods). The identified methylation motifs were summarized per isolate and classified to their type, as reviewed in (33). Methylation motifs highlight methylated bases in green, red, or purple for 6mA, 5mC, and 4mC methylated bases, respectively. Only one motif orientation is presented for type II and palindromic type I motifs. Only motifs with at least 100 genomic occurrences are shown, except for the 4mC motif of PA188 (*). The identification and classification of DNA methyltransferases (MTases) for each genome are also summarized in the same table. Shades of blue represent abundances across all values. (**B**) In-depth analysis of discrepancies of *P. aeruginosa* isolates PA068 (46 discordant positions, one methylation motif [CA**5mC**GTG], and four MTases with unknown motif) and PA188 (25 discordant positions, one methylation motif [CGC**4mC**GGG], and three MTases of which one type II MTase with known motif [TGGCC**6mA**]) in comparison with PA200 (two discordant positions, five methylation motifs [with GAAG**6mA**G and **4mC**TCTTC], and 13 MTases). of which three with known motif) and PA075 (fully concordant genome, four methylation motifs, and 13 MTases of which five with known motif). For both PA068 and PA188 isolates, the positions within and around GGCC occurrences are shown in the logo plots. For isolate PA200, the same was observed but centered around its GAAGAG motif. All positions with piled read mQ-scores dropping <10 were plotted with dark blue lines for each isolate, representing 1,716, 1,646, and 77 reads for PA068, PA188, and PA200, respectively. A random subsample of the same size of regions with adequate quality (overall mean Q-score of 23) with GGCC or GAAGAG motif is plotted in light blue for comparison. Logo plots were obtained from the same plotted genomic regions spanning a total of 20 nucleotides. Isolate PA075 was included as a high-quality reference not showing any drops with mQ-score <10 for which 1,000 random sequences were selected and plotted.

Since we aimed to link the optimized genome assembly workflow to an open-source cgMLST analysis, we also performed a cgMLST analysis on the test data set using pyMLST (13). This analysis demonstrated high concordance between cgMLST allele distances obtained from SRS and LRS genome assemblies (Fig. 2C and Zenodo). While completely concordant cgMLST distances were observed for eight of the ten isolates, *P. aeruginosa* isolates PA068 and PA188 demonstrated 27 and 15 allelic differences between the SRS and LRS, respectively. These two *P. aeruginosa* test isolates also demonstrated increased numbers of total discordant positions (46 and 25 for PA068 and PA188, respectively) for all tested ONT-only pipelines (Fig. 2A and B). Evaluation of these positions revealed that nearly all discrepancies arise at GGCC motifs (Table S3 and Zenodo). To establish whether methylation at this position might be the cause of these erroneous basecalls, genome-wide analysis of methylation was performed. Notably, methylation events were not consistently identified within either of the genomes in association with this motif (Fig. 3A and Zenodo). For isolate PA188, a 4mC methylation motif was predicted (CGC4mCGGG) at low prevalence, along with a predicted type II DNA methyltransferase (MTase) that could methylate a TGGCC6mA motif. Overall, both isolates PA068 and PA188 demonstrated lower numbers of predicted methylation motifs and DNA MTases compared to the other isolates (Fig. 3A). A more detailed analysis revealed a clear drop in raw read mean Q-score (defined as mQ-score <10, as compared to overall mQ-score of 23; Table S1) at 1,716 and 1,646 GGCC genomic positions containing the 46 and 25 residual discordant positions for PA068 and PA188, respectively (Fig. 3B).

Investigation of the sequence context around the GGCC motif failed to reveal a common extended motif, though a small population of G-A substitutions was observed at the second position within the GGCC motif, representing the majority of discordant positions (Fig. 3B). Similar motif-associated drops in quality scores were also observed for *P. aeruginosa* PA200 but at a lower frequency (*n* = 77), whereas they were not obviously apparent in the eight other genomes (PA075 is included as a representative example in Fig. 3B). For PA200, methylation motif prediction revealed a GAAG6mAG methylation together with a 4mCTCTTC methylation on its opposite strand. While an overall drop in mQ-scores was observed at different genomic positions for this “mixed” motif, the two residual adjudicated ONT errors were attributable to drops in mQ-score below 10. The same observation was made for the GGCC positions for which only a fraction (46/1,716 (70,459 total) and 25/1,646 (83,098 total) for PA068 and PA188, respectively) had residual discordant positions (Fig. 3; Table S3 and Zenodo). Of note, one genome showed a slight decrease in the number of discordant positions (25 to 21 for PA188) with the application of medaka polishing with corrected reads, indicating successful removal of some of these GGCC-mediated errors upon raw read error correction and subsequent polishing (Fig. 2A). Importantly, six out of ten test *P. aeruginosa* isolate genomes were fully concordant when compared to respective SRS data sets. Another two demonstrated a maximum of two residual discordant positions in the final LRS-only genomes, which did not impact cgMLST analysis (Fig. 2).

### Nanopore-only sequencing can generate cgMLST minimum spanning trees fully concordant with Illumina analysis

**Fig 4:**
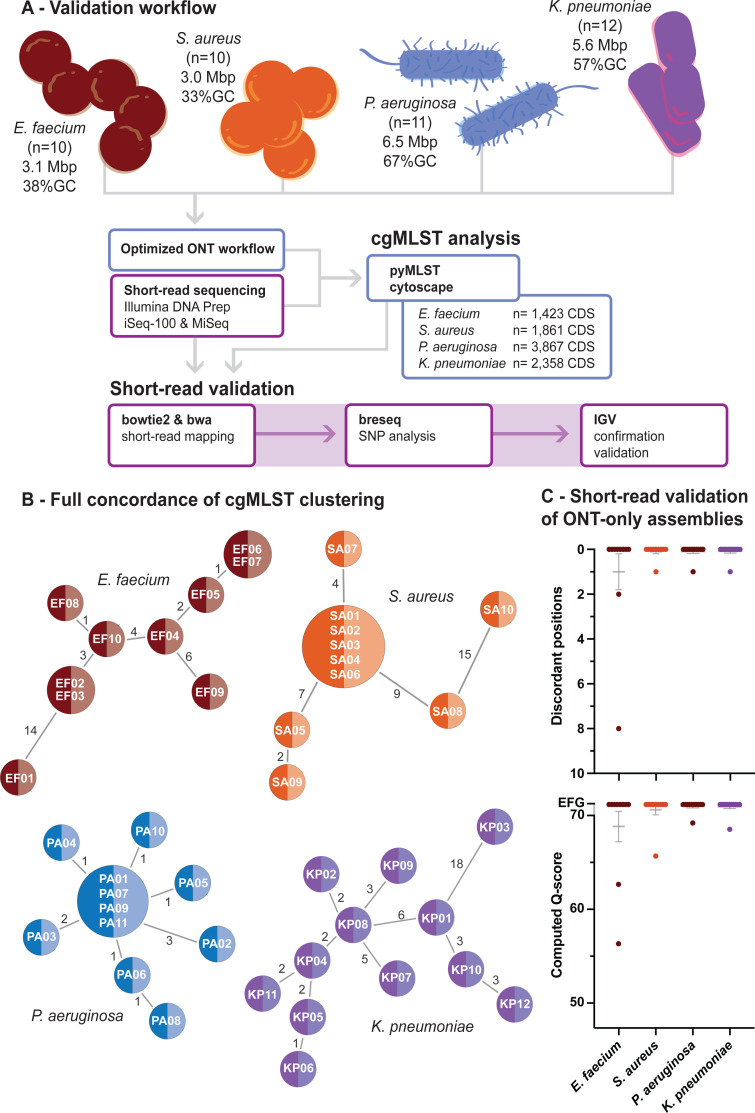
Validation of the optimal ONT-only workflow on four outbreak clusters demonstrating full concordance. (**A**) Schematic overview of the validation workflow using four hospital outbreak clusters. Four bacterial pathogens commonly identified in hospital outbreaks were selected for pipeline validation. All isolates (*n* = 43), including *E. faecium*, *S. aureus*, *P. aeruginosa*, and *K. pneumoniae*, were subjected to the optimized ONT workflow, followed by independent Illumina short-read sequencing from the same HMW gDNA. cgMLST minimum spanning trees were generated and compared with the short-read reference using breseq followed by confirmation/validation in IGV (Tables S4 and S5, and Methods for classification of discordant positions). (**B**) Short-read cgMLST validation analysis showing fully concordant cgMLST minimum spanning trees and pairwise distances for four hospital outbreak clusters, including *E. faecium* (*n* = 10; brown), *S. aureus* (*n* = 10; orange), *P. aeruginosa* (*n* = 11; blue), and *K. pneumoniae* (*n* = 12; purple). Minimum spanning trees were generated after short-read validation and classification of all discordant SNPs (methods), with branch lengths representing the number of allelic differences. Dark and light colors represent ONT and short-read data sets, respectively. (**C**) Chromosomal discordant positions (upper panel) and computed Q-score (lower panel) for four ONT-only assembly hospital outbreak clusters with discordant positions calculated based on the short-read data sets. Computed Q-scores have varying species-specific ranges due to dependence on the genome size. Mean ± s.e.m. are plotted for each bacterial species with individual data points in respective colors; EFG, error-free genomes based on Illumina references, corresponding to 0 discordant positions after applying exclusion criteria.

To test the performance of the optimized workflow, we subjected four hospital outbreak clusters to the streamlined ONT-only workflow and parallel SRS from the same extracted HMW DNA (Fig. 4A). For this analysis, *Klebsiella pneumoniae* (*n* = 12), *P. aeruginosa* (*n* = 11), *Enterococcus faecium* (*n* = 10), and *Staphylococcus aureus* (*n* = 10) were chosen to represent gram-negative and gram-positive bacteria over a range of genomic GC contents. Additionally, these isolates spanned cgMLST distances computed from short reads ranging from 0 to 18 allelic differences to cover highly clonal and epidemiologically unrelated (but still similar) isolates for realistic simulation. Application of the optimized workflow generated minimum spanning cgMLST trees for all four sets that were fully concordant with the Illumina SRS data from the same HMW DNA (Fig. 4B). To assess overall concordance between SRS and LRS whole-genome assemblies, validation was performed using the ONT-only genomes and SRS data set for each isolate. This analysis independently confirmed the high concordance between the optimized ONT approach and Illumina SRS and demonstrated at the maximum two discordant chromosomal positions in 42/43 genomes (98%). The only exception was one *E. faecium* isolate EF09 (Fig. 4C; Table S4), which had eight discordant positions that were mostly indels associated with genomic homopolymer regions (Table S4 and Zenodo). Importantly, *E. faecium* EF09 was the only isolate subjected to Flongle sequencing, which may have contributed to the greater number of discordant positions. Still, no discordant cgMLST alleles were observed compared to the SRS reference for this isolate (Fig. 4B). These results are in line with the observations made with the *P. aeruginosa* test set and demonstrate the broader applicability of the optimized workflow across four relevant bacterial pathogens with varying genome size and GC content.

## DISCUSSION

In this study, we have optimized a start-to-finish nanopore-only workflow for cgMLST analysis in the context of bacterial clonality detection and outbreak tracing. We chose to begin workflow optimization with a *P. aeruginosa* isolate test set, which permitted the assessment of a rapid barcoding library preparation strategy for high-GC content organisms. Lower tagmentation efficiency and uniformity have been reported for high-GC content DNA with both PCR-based and PCR-free ONT rapid library barcoding kits (1, 16, 21, 22, 34). In this work, we demonstrated significant improvements in rapid barcoding library efficiency and throughput with the simple implementation of a tagmentation strategy that incorporates temperature ramps, which may improve access to DNA structures that form in regions of high GC content. Along with improvements in efficiency, a small reduction in median read Q-scores was observed with the application of this protocol. This may have a few different potential origins, including more challenging sequencing content (e.g*.,* complex GC repeats) incorporated into the ramped library preparations, but this was not investigated further. The minor increase in the error rate was not found to interfere with the generation of highly concordant genome assemblies in this study. This small adjustment to the existing rapid barcoding protocol may facilitate a broader application for pathogen surveillance of more diverse bacterial taxa (e.g*.,* Burkholderiaceae [16]) without the need for more extensive ligation-based library preparations.

Using sequencing data from the test data set, we performed extensive benchmarking analysis, which demonstrated improvements resulting from the dorado basecalling model sup@v5.0.0 and its read-correcting HERRO algorithm, followed by a standard *de novo* Flye-based genome assembly workflow and post-assembly polishing with dorado. Raw read error correction resulted in genome assemblies of similar quality when compared to assemblies obtained from uncorrected reads and polished with medaka or dorado polish. We did not observe meaningful differences between medaka (v2.0.1) or dorado polishing with their bacterial models, which may be expected, as the tested versions of both tools apply the same models (10.5281/zenodo.14829720). It should be noted that at the time of this writing (April 2025), medaka is the preferred polisher recommended by ONT for bacterial WGS (31, 32). However, we chose to include a single open-source tool for basecalling, demultiplexing, error correction, read mapping, and polishing in the workflow we present to reduce potential difficulties brought about by complex software dependencies and compatibilities. Taking the above into consideration, the final pipeline consisted of dorado sup@v5.0.0 basecalling, dorado error correction, Flye *de novo* genome assembly, and dorado polish with its bacterial model. It should be noted that care should be taken when using dorado correct (v0.9.1) within this pipeline as the algorithm (i) filters all reads shorter than 4 kb; (ii) may result in the removal of low-frequency variants/alleles; (iii) can split reads when support/coverage is too low (30). In addition, we strongly encourage users of this optimized workflow to stay current with updated versions of the included tools, especially those released by ONT (e.g*.,* dorado and medaka). Frequent releases of new basecalling models have contributed to swift changes and improvements in raw read accuracies and should be followed closely.

It is important to note that in our analysis we have focused only on chromosomal assembly and have excluded plasmids as the primary focus was clonality analysis. It should be kept in mind that the recovery of small plasmids generally imposes a challenge for many long-read assemblers, including the programs in the tools proposed here (35). If extrachromosomal elements (e.g*.,* plasmids or phages) are of importance, more extended genome assembly tools such as Trycycler and its new automated counterpart, Autocycler (36), can be considered for implementation in the current workflow (27). Since the goal of this study was to deliver a streamlined workflow focusing on downstream cgMLST analyses, a simple Flye-based assembly was chosen due to its potential for wider implementation. While the most concordant consensus genomes can be obtained with raw read error correction, remapping of original uncorrected reads can be performed for downstream analyses involving the study of subgenomic variants (e.g*.,* structural or SNPs). Additionally, although the current study did not include analyses of the use of LRS for the investigation of antimicrobial resistance and virulence-associated genes, including those encoded on plasmids, increased accuracy of consensus genomes obtained with the optimized workflow would improve identification of SNPs contributing to antimicrobial resistance (1, 37, 38).

An important question in all genome sequencing and analyses is how much sequencing depth is required for accurate and complete assemblies. The answer to this question depends in detail on error distributions following correction and polishing and how they resolve in the consensus assembly. We asked this question with a subsampling analysis performed on the final pipeline with dorado correct and polish. We found that a minimum sequencing depth of ~50 x was required to achieve highly concordant genomes in this analysis. This conclusion is in line with previous reports of “older” ONT-only workflows and indicates that moderate depth sequencing with this workflow is sufficient for many applications (1, 21).

The choice of a *P. aeruginosa* test data set in our benchmarking and subsampling analyses allowed us to assess the robustness of the computational pipeline on a bacterial species that historically caused trouble for both SRS and LRS due to its high genomic GC content. The optimized ONT-only workflow demonstrated fully concordant cgMLST distances for eight out of ten isolates, with all eight demonstrating a maximum of two adjudicated ONT errors within their 6.5 Mb genomes after the applied quality exclusions. The two other isolates demonstrated a greater number of discordant positions, which were attributed to lower raw read mean Q-scores within a minor fraction of genomic GGCC sequences (Fig. 3 and Methods). Previous analyses have demonstrated that a sizeable proportion of basecall errors are likely due to methylation, which can be highly divergent within and across bacterial taxa (1, 20, 21, 23, 25, 33), though no putative methylation motifs that could consistently explain basecall errors in these two *P. aeruginosa* test isolates were identified. Delahaye and Nicolas (22) reported a 1.5% increased mismatch error rate, largely due to G-A substitutions, for high GC organisms, as compared to those with lower GC content in data from the retired R9.4.1 flow cell chemistry and guppy basecalling (22). This is in line with our observations that residual GGCC motif-related errors appear to result in G-A substitutions, producing GACC erroneous calls (Fig. 3). Surprisingly, both isolates had lower numbers of predicted methylation motifs and DNA MTases encoded within their genomes when compared to the well-performing genomes (Fig. 3A). This relationship suggests a potential “overcorrection” when using current methylation-aware and all-context models, which is in line with a recent report on *Burkholderia pseudomalei* nanopore-only genome assemblies (16). Whether these recent methylation-aware and all-context basecalling and polishing models impact accuracy for bacterial genomes with reduced or less complex methylation events will require further investigations. These could also be base modifications of a yet unknown nature or multiple rare methylation events within a single k-mer for which these models have not yet been trained. The possibility for such errors should thus be kept in mind when applying the presented pipeline to high GC content genomes. What would be considered an “acceptable” number of discordant positions would depend on the thresholding that is being used for the particular cgMLST scheme. As the number of discordant positions (due to true ONT sequencing errors) approaches this threshold, it will be expected to impact clonality assessment. Standard cgMLST schemes use thresholds that range from 5 to 30, and thus whole-genome error rates must generally remain below this range to be useful for epidemiologic analysis (39–42).

Assessment of the final optimized workflow on a validation set consisting of *K. pneumoniae*, *P. aeruginosa*, *E. faecium*, and *S. aureus* outbreak isolates demonstrated fully concordant (identical) cgMLST-based minimum spanning trees for all four sets when compared to the SRS reference standards. In addition, whole genomes could be assembled with a maximum of two discordant positions in 42/43 genomes when compared to the SRS reference following the quality exclusions described here. This indicates that with proper computational analysis, ONT-only sequencing can be used for clonality analysis and outbreak tracing, representing a milestone for nanopore sequencing. We believe that access to nanopore sequencing devices and streamlined open-source pipelines such as the one presented here will facilitate implementation of nanopore-only clonality and outbreak analysis in the context of public health and clinical microbiology, contributing critically to hospital infection control.

## MATERIALS AND METHODS

### Bacterial isolate selection and growth conditions

For test optimization, a set of ten de-identified, unrelated clinical *P. aeruginosa* isolates were obtained from the National Institutes of Health Clinical Center (National Institutes of Health, Bethesda, MD). The test isolates were cultivated from frozen stocks on LB agar plates up to 24 h at 37°C prior to DNA extraction, as described below. For testing of the optimized pipeline, a validation isolate set was compiled from the Walter Reed Army Institute of Research Multi-Drug-Resistant Organism Repository and Surveillance Network (WRAIR-MRSN, Silver Spring MD). This validation set comprised de-identified patient and environmental isolates from four outbreak clusters: *Staphylococcus aureus* (*n* = 10), *Enterococcus faecium* (*n* = 10), *P. aeruginosa* (*n* = 11), and *Klebsiella pneumoniae* (*n* = 12). All validation isolates were grown on tryptic soy agar (TSA; Remel) stabs up to 24 h at 37°C.

### Extraction of High-Molecular Weight DNA

Bacterial colonies were collected from agar plates or stabs and resuspended in 1 mL phosphate-buffered saline (PBS, pH 7.4; Invitrogen) for DNA extraction. HMW DNA was extracted using the Maxwell HT gDNA Blood kit (Promega) on a Kingfisher Flex system (ThermoFisher). For gram-positive isolates, an additional lysozyme treatment (10 mg.mL^−1^; MP Biomedicals) was performed for 30 minutes at 37°C prior to extraction. After HMW DNA extraction, all samples were subjected to an additional RNase A treatment (80 µg.mL^−1^; Promega) and purified using a 1:1 AmPure XP bead (Beckman Coulter) cleaning protocol with freshly prepared 80% ethanol. Initial quality assessment of the resulting DNA was performed with a Nanodrop One (ThermoFisher), followed by DNA quantification and integrity assessment using the Qubit dsDNA BR assay kit (Invitrogen) and TapeStation gDNA Screentapes on a 4200 TapeStation (Agilent Technologies). Quality assessment of DNA is summarized in Table S1, with additional files uploaded to Zenodo (10.5281/zenodo.15103235).

### Rapid barcoding strategy incorporating temperature ramps for high-GC content genomes

Four *P. aeruginosa* isolates from the test set (isolates PA075, PA099, PA200, and PA223) were chosen to evaluate a modified ONT rapid barcoding library preparation strategy incorporating temperature ramps to improve the performance on high-GC content genomes. For this assessment, libraries were prepared from the HMW DNA from each isolate in parallel using the same barcode sets to compare both approaches. One library for each set was prepared using the standard rapid barcoding kit (SQK-RBK114.24; ONT) as per the manufacturer’s instructions, and the other was prepared using a modified version incorporating a slow temperature ramp between the transposon cleavage and inactivation step (0.1°C.s^−1^ from 30°C to 80°C). These libraries were loaded onto individual R10.4.1 Flongle flow cells (FLO-FLG114; ONT) and sequenced for 24 h. Data were collected within the MinKNOW software (v.24.02.16; ONT; dorado sup@4.3.0), and sequencing statistics were generated by NanoComp (v1.23.1 [43]). Since sequencing performance involving different flow cells depends on the number of available pores, the number of reads and sequencing throughput were normalized to the maximum number of available pores per Flongle. Sequencing statistics are summarized in Table S2.

### Library preparation and ONT sequencing of test set and validation sets

ONT nanopore sequencing libraries were prepared from HMW DNA from all test and validation set isolates using the rapid barcoding kit (SQK-RBK114.24; ONT). Validation set *S. aureus*, *E. faecium*, and *K. pneumoniae* isolates were processed following the manufacturer’s instructions. The *P. aeruginosa* libraries for the test and validation sets were prepared using the modified approach incorporating temperature ramps, as described above. All samples belonging to the test and validation sets were multiplexed per species and sequenced on R10.4.1 GridION/MinION flow cells up to 72 h (FLO-MIN114; ONT). MinION data for *E. faecium* EF09 were substituted with additional R10.4.1 Flongle sequencing due to lowered sequencing throughput as a result of barcode imbalance. All final sequencing libraries were prepared and loaded according to the manufacturer’s instructions with sequencing on a GridION system (ONT) and real-time data collection in MinKNOW (v.24.02.16; ONT). Sequencing statistics were obtained from NanoComp and are summarized in Table S1.

### Benchmarking and subsampling of ONT basecalling, error correction, and draft genome polishing pipelines with the *P. aeruginosa* test set

The benchmarking approach focused on the comparison of dorado (v0.9.1; ONT) error correction, genome polishing algorithms, and associated models within a standard genome assembly workflow. Basecalling and demultiplexing were done using the dorado basecaller applying either of its super-accuracy models (sup@v4.3.0 or sup@v5.0.0), followed by read quality filtering with chopper (--quality 10; v0.9.1 [43]). While the basecalled reads were used directly for draft assembly, the sup@v5.0.0 were also subjected to raw read error correction using dorado correct (with its herro-v1 model [30]). Of note, using dorado correct at the time of this analysis (April 2025), a small fraction of split reads was observed in its output file, possibly due to low coverage allelic variants. These reads were flagged as 1–5 in their fasta header, allowing removal from the final output. Both uncorrected and corrected reads were used in the standard *de novo* genome assembly workflow with Flye (v2.9.5; --nano-hq [44]), followed by reorientation of contigs using dnaapler (v1.2.0; github.com/gbouras13/dnaapler). Resulting drafts were subjected to polishing, for which reads were realigned to their respective assemblies. Genomes were polished with medaka (v2.0.1; --bacteria using the r1041_e82_400bps_bacterial_methylation model; ONT) using either uncorrected (sup@v5.0.0) or corrected reads that were aligned using minimap2 (v2.28 [45]) and samtools (v1.19; -bS -F 2308 [46]). In parallel, genome polishing was done with dorado polish (--bacteria, using the dna_r10.4.1_e8.2_400bps_polish_bacterial_methylation_v5.0.0 model), for which the raw sup@v5.0.0 reads were aligned to the drafts using dorado aligner, which is required to include move tables (i.e*.,* dwell times) in the alignment files. An overview of this benchmarking workflow is presented in Fig. 1, in which the optimal workflow is highlighted in dark blue. Of note, the sup@v4.3.0 reads were only used to set a genome assembly baseline using the reads in the same Flye-based genome assembly workflow, followed by medaka (v2.0.1; --bacteria) draft polishing with minimap2-remapped sup@v4.3.0 reads. Finally, the minimum required LRS depth was assessed for the optimized workflow by subsampling the test data set at depths ranging between 10 and 160 x using samtools view (--subsample). If a sample did not have sufficient coverage for a subsampling depth, it was excluded from the analysis (Fig. 1 light blue). Genome completeness of the most accurate genome assemblies was determined with CheckM2 (v1.1.0 [47]), with all LRS and assembly statistics summarized in Table S1 and reports available on Zenodo (10.5281/zenodo.15103235). All analyses were performed with default settings, unless stated otherwise.

### Short-read sequencing and assembly

Illumina sequencing libraries were prepared from HMW DNA using Illumina DNA prep libraries (Illumina) with 100–300 ng starting material, 5 PCR cycles, and IDT for Illumina UD Indexes (Illumina). Libraries of nine of the ten *P. aeruginosa* test isolates were prepared from a prior DNA extraction, i.e*.,* not the same one as used for ONT sequencing. Libraries for *P. aeruginosa* test set isolate PA228-2 and all validation set isolates were prepared from the same HMW DNA as used for ONT sequencing. Libraries were sequenced on a combination of iSeq-100, MiSeq, and NextSeq 2000 systems. Sequencing was done with the exclusion of any read sets not meeting applied quality standards. All reads were filtered using Trimmomatic (v0.39; ILLUMINACLIP:NexteraPE-PE.fa:2:30:10:2:True SLIDINGWINDOW:4:20 LEADING:30 TRAILING:30 HEADCROP:25 MINLEN:50 [48]), followed by the SPAdes (v3.15.5; --isolate -m 60 [49]) assembly. The quality of reads was assessed using FastQC (v0.12.0; https://www.bioinformatics.babraham.ac.uk/projects/fastqc/) and multiQC (v1.27.1 [50]). Genome completeness of the SRS assemblies was determined with CheckM2 (v1.1.0 [47]), with all SRS and assembly statistics summarized in Table S1 and reports available on Zenodo (10.5281/zenodo.15103235). All analyses were performed with default settings, unless stated otherwise.

### Comparison of ONT-only assemblies with Illumina reference

To assess concordant positions between long-read ONT-only assemblies within both test and validation data sets, initial SRS validation was done within the breseq suite (v0.37.1 [51]) applying default settings. Note that the validation isolates first underwent ONT sequencing and were analyzed with the final optimized pipeline in a manner blinded to the SRS data. Resulting discordant positions were further investigated by remapping short- and long-read data sets to their corresponding ONT-only assembly using bwa2 (v2.2.1 [52]) or minimap2, respectively. Alignment files were created using samtools (-bS -F 2308), allowing manual inspection and curation of all discordant positions in IGV (v2.16.2 [53]) (Table S3). To report adjudicated ONT errors relative to the Illumina reference, all discordant positions were classified into one of six categories, with the retention of only adjudicated ONT errors for final SRS validation. These six categories included the following: (i) adjudicated ONT errors; (ii) discordant position located on a non-chromosomal contig (e.g*.,* plasmids); (iii) discordant position associated with a drop in SRS coverage*,* defined as <25% of median genome coverage, as often seen in GC-rich regions or repeats; (iv) discordant position due to multi-allelic variants confirmed in both SRS and LRS data sets; (v) discordant position as a result of the presumed error in the SPAdes genome assembly algorithm and not supported by SRS raw read remapping; (vi) discordant position due to misalignment of extrachromosomal elements (e.g*.,* plasmids and insertion sequences) in either SRS and LRS data sets. An overview of these filtering criteria is presented in Fig. S1, and all discordant positions and their classification are summarized in Tables S3 and S4 with IGV snapshots on the Zenodo repository (10.5281/zenodo.15103235). Adjudicated ONT errors were then used to compute Q-scores for each of the test and validation data sets, as presented in Table S5, using the Q-score formula and genome sizes as in Fig. S1.

An in-depth LRS methylation analysis was then performed on the test data set to assess the potential contributions of methylation events to adjudicated ONT errors, as classified in (i) above. Here, pod5 files were basecalled again using the dorado basecaller and its 6mA and 4mC_5mC methylation models (v3 within sup@v5.0.0; ONT). Resulting bam files were used to generate methylation pileups using modkit (v0.4.1; ONT) with its respective optimal ONT-only genome assembly as the reference. Final bedmethyl files were used to predict methylation motifs with modkit motif search (--min-sites 100), along with the prediction of DNA methyltransferases (MTase) using DNA_methylase_finder (github.com/mtisza1/DNA_methylase_finder [54]). All predictions are available on Zenodo (10.5281/zenodo.15103235). An in-depth motif analysis was performed for *P. aeruginosa* isolates PA068, PA188, and PA200 with the inclusion of isolate PA075 as a representative error-free reference. First, basecall quality mean Q-scores (mQ-score) were obtained from alignment files at every genomic position with pysam (v0.23.0; github.com/pysam-developers/pysam). Positions with mQ-score <10 were identified with the find_peaks() function in scipy (v.1.15.2 [55]) and were compared with background sequences. For PA068 and PA188, an equal number of random GGCC occurrences was used as the background, whereas for PA200, all occurrences of the GAAGAG motif were used. For PA075, 1,000 random regions were included as a representative example. Finally, all positions were plotted with Matplotlib, and sequence contexts were extracted for the drops and used to create motif logo plots with WebLogo (v2.8.2 [(56]). All analyses were performed with default settings, unless stated otherwise.

### Open-source cgMLST analysis using pyMLST

The open-source tool pyMLST (v2.1.6 [13]) was used to perform cgMLST with published schemes available through cgMLST.org (6–10). The SRS and optimal LRS genome assemblies were analyzed as individual entries to assess cgMLST concordance between the SRS and LRS assemblies. The pyMLST distance subcommand was used to calculate the number of allelic differences between all isolates. Missing targets in any of the assemblies, because of absence or incomplete genes at contig borders, were excluded from this analysis. All allelic differences between SRS and LRS assemblies were further validated and filtered as described above, retaining only deemed true discordant positions in the cgMLST distance matrix. Final distance matrices were then used to obtain minimum spanning trees with the minimum_spanning_tree() function in networkx (v3.4.2 [57]), followed by visualization in Cytoscape (v3.10.3 [58]) and Adobe Illustrator (v29.3.1). All distance matrices and associated cgMLST tables are available on Zenodo (10.5281/zenodo.15103235) and in Tables S3 and S4.

### Statistical analysis and data visualization

Statistical analysis was performed in GraphPad Prism (v10.4.1) using a paired *t*-test for which significance was considered when *P* < 0.05. Data are presented as mean ± s.e.m. and were visualized in GraphPad Prism and Adobe Illustrator.

## Data Availability

The raw sequencing data generated in this study have been deposited in the NCBI database under BioProject accession code PRJNA1255637. All other relevant data files are presented in either supplementary tables or figures or can be found on Zenodo (10.5281/zenodo.15103235).

## SUPPLEMENTAL MATERIAL

Figure S1 jcm.00664-25-s0001.pdf Schematic overview of the applied short-read validation strategy with respective classification of all discordant positions.

Supplemental tables jcm.00664-25-s0002.xlsx Tables S1 to S5.

ASM does not own the copyrights to Supplemental Material that may be linked to, or accessed through, an article. The authors have granted ASM a non-exclusive, world-wide license to publish the Supplemental Material files. Please contact the corresponding author directly for reuse.
