# Supplementary material for: An open-source nanopore-only sequencing workflow for analysis of clonal outbreaks delivers short-read level accuracy
Source: J Clin Microbiol. 2025 Jul 18;63(8):e00664-25. doi: 10.1128/jcm.00664-25 (PMC12345217; doi:10.1128/jcm.00664-25)
Supplement: Figure S1 — Schematic overview of the applied short-read validation strategy with respective classification of all discordant positions. [file jcm.00664-25-s0001.pdf]

An open-source nanopore-only sequencing workflow for analysis of clonal outbreaks delivers short-read level accuracy

Nick Vereecke[1], Thomas B. Yoon[1], Ting L. Luo[2], Brendan W. Corey[2], Francois Lebreton[2], Patrick T. Mc Gann[2], and John P. Dekker[1*]

[1] Bacterial Pathogenesis and Antimicrobial Resistance Section (BPARS), Laboratory of Clinical Immunology & Microbiology (LCIM), National Institute for Allergy and Infectious Disease (NIAID), National Institutes of Health (NIH), Bethesda MD; [2] Multidrug-Resistant Organism Repository and Surveillance Network (MRSN), Diagnostics and Countermeasures Branch, Center for Infectious Disease Research (CIDR), Walter Reed Army Institute of Research (WRAIR), Silver Spring MD

* Corresponding author (john.dekker@nih.gov)

Supplementary Figure

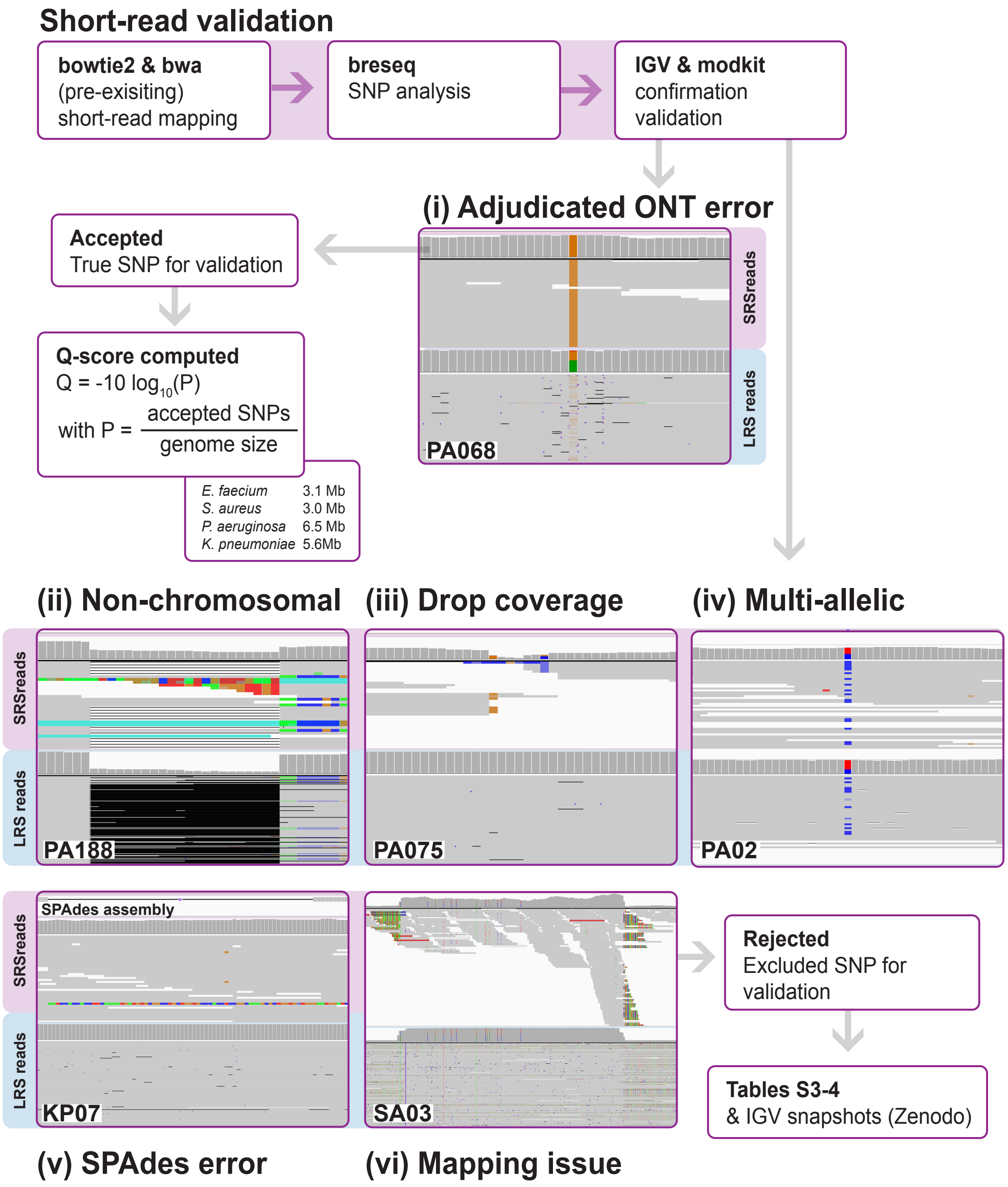

FIG S1 Schematic overview of the applied short-read validation strategy with respective classification of all discordant positions. Each reported discordant position (as obtained from breseq) was manually confirmed and validated by remapping both SRS and LRS datasets

to the respective ONT LRS assembled genome. Each discordant position was then classified into one of six discordant groups: (i) adjudicated ONT error, (ii) discordant position located on a non-chromosomal contig (*e.g.,* plasmids), (iii) discordant position due to of a drop in SRS coverage, defined as <25% of median genome coverage, (iv) discordant position due to multi-allelic variants confirmed in both SRS and LRS datasets, (v) discordant position due to errors (*i.e.,* SNPs or structural variants) in the SPAdes genome assembly algorithm and not confirmed in SRS remapping, and (vi) discordant position due to misalignment of extrachromosomal elements (*e.g.,* plasmids and insertion sequences) in either or both SRS and LRS datasets. For each category a representative IGV snapshot example is given along with the isolate name (PA068: chromosomal position 4,808,372; PA188: plasmid position 21,292; PA075: chromosomal position 1,995,050; PA02: chromosomal position 3,453; KP07: chromosomal position 8,385; SA03: chromosomal position 35,544). The SRS (transparent purple) and LRS (transparent blue) datasets are presented at top and bottom of each IGV snapshot, with the inclusion of the SPAdes assembly for category v. Only adjudicated ONT errors (category i) were accepted for validation purposes and used for cgMLST and wgSNP analyses, along with computation of Q-scores. All other discordant positions (*i.e.,* category ii-vi) were rejected for validation and were summarized in **Tables S3-4** along with all IGV snapshots on Zenodo (10.5281/zenodo.15103235).
